# Elevated serum galectin-1 concentrations are associated with increased risks of mortality and acute kidney injury in critically ill patients

**DOI:** 10.1371/journal.pone.0257558

**Published:** 2021-09-24

**Authors:** Ruey-Hsing Chou, Chuan-Tsai Tsai, Ya-Wen Lu, Jiun-Yu Guo, Chi-Ting Lu, Yi-Lin Tsai, Cheng-Hsueh Wu, Shing-Jong Lin, Ru-Yu Lien, Shu-Fen Lu, Shang-Feng Yang, Po-Hsun Huang

**Affiliations:** 1 Division of Cardiology, Department of Medicine, Taipei Veterans General Hospital, Taipei, Taiwan; 2 Cardiovascular Research Center, National Yang Ming Chiao Tung University, Taipei, Taiwan; 3 Department of Critical Care Medicine, Taipei Veterans General Hospital, Taipei, Taiwan; 4 Institute of Clinical Medicine, National Yang Ming Chiao Tung University, Taipei, Taiwan; 5 Department of Medical Research, Taipei Veterans General Hospital, Taipei, Taiwan; 6 Taipei Heart Institute, Taipei Medical University, Taipei, Taiwan; 7 Division of Cardiology, Heart Center, Cheng-Hsin General Hospital, Taipei, Taiwan; 8 Department of Nursing, Taipei Veterans General Hospital, Taipei, Taiwan; 9 School of Nursing, National Yang Ming Chiao Tung University, Taipei, Taiwan; 10 Division of Nephrology, Department of Medicine, Cheng Hsin General Hospital, Taipei, Taiwan; Asan Medical Center, University of Ulsan College of Medicine, REPUBLIC OF KOREA

## Abstract

**Background:**

Galectin-1 (Gal-1), a member of the β-galactoside binding protein family, is associated with inflammation and chronic kidney disease. However, the effect of Gal-1 on mortality and acute kidney injury (AKI) in critically-ill patients remain unclear.

**Methods:**

From May 2018 to March 2020, 350 patients admitted to the medical intensive care unit (ICU) of Taipei Veterans General Hospital, a tertiary medical center, were enrolled in this study. Forty-one patients receiving long-term renal replacement therapy were excluded. Serum Gal-1 levels were determined within 24 h of ICU admission. The patients were divided into tertiles according to their serum Gal-1 levels (low, serum Gal-1 < 39 ng/ml; median, 39–70 ng/ml; high, ≥71 ng/ml). All patients were followed for 90 days or until death.

**Results:**

Mortality in the ICU and at 90 days was greater among patients with elevated serum Gal-1 levels. In analyses adjusted for the body mass index, malignancy, sepsis, Sequential Organ Failure Assessment (SOFA) score, and serum lactate level, the serum Gal-1 level remained an independent predictor of 90-day mortality [median vs. low: adjusted hazard ratio (aHR) 2.11, 95% confidence interval (CI) 1.24–3.60, *p* = 0.006; high vs. low: aHR 3.21, 95% CI 1.90–5.42, *p* < 0.001]. Higher serum Gal-1 levels were also associated with a higher incidence of AKI within 48 h after ICU admission, independent of the SOFA score and renal function (median vs. low: aHR 2.77, 95% CI 1.21–6.34, *p* = 0.016; high vs. low: aHR 2.88, 95% CI 1.20–6.88, *p* = 0.017). The results were consistent among different subgroups with high and low Gal-1 levels.

**Conclusion:**

Serum Gal-1 elevation at the time of ICU admission were associated with an increased risk of mortality at 90 days, and an increased incidence of AKI within 48 h after ICU admission.

## Introduction

Galectin 1 (Gal-1) is a member of the galectin family that has one carbohydrate recognition domain. It is expressed throughout the body and involved in T-cell homeostasis, which in turn regulates the immune response and host–pathogen interaction [1]. Recombinant Gal-1 is expressed to ameliorate acute and chronic inflammation in patients with autoimmune encephalomyelitis and those with collagen-induced arthritis [2–4]. Gal-1 is reported to be the mediator of cardiovascular inflammation [5]. It also mediates the interaction of cancer cells with the extracellular matrix [6]. Numerous literatures indicate the regulatory functions of Gal-1 in viral [7], bacterial [8], and parasite infection [9]. One recent review even suggested the galectins to have therapeutic value in treating influenza A virus infection [10]. However, there is currently no clinical evidence supports the association between Gal-1 and sepsis. Gal-1’s role in critically ill patients remains poorly understood.

Sepsis, characterizes by uncontrolled infection and life-threatening organ dysfunction, is the leading causes of death among critically ill patients in medical ICUs [11]. Even non–infection-related systemic inflammatory response syndrome (SIRS) carries similar mortality risks in patients in the intensive care unit (ICU) [12]. In a previous study, 88.4% of patients in the ICU had at least two criteria of SIRS [13]. In this single-center observational study, we examined associations of the serum Gal-1 level with all-cause mortality and acute kidney injury (AKI) in septic or critically ill patients admitted to the ICU. The aim of this study is to investigate relationship between circulating Gal-1 and mortality in critically ill patients, and the primary outcome is all-cause 90-days mortality. We hypothesized that patients with higher Gal-1 concentrations would have higher incidences of mortality.

## Materials and methods

### Study population

This study was carried out in the medical ICU of Taipei Veterans General Hospital, a tertiary medical center in Taipei, Taiwan. From May 2018 to March 2020, we prospectively screened 350 patients admitted to the medical ICU because of various critical illnesses, such as sepsis, pneumonia, massive gastrointestinal bleeding, and acute heart failure. Sepsis and septic shock were defined according to the 2016 Surviving Sepsis Campaign guidelines [14]. Patients with pre-dialysis status before ICU admission (*n* = 41) were excluded from analysis. Patients were admitted from the emergency department or transferred from the ordinary ward. In total, 309 patients provided informed consent and participated in this study. Confounding factors that were reported to be associated with ICU mortality [15, 16], AKI [17], or serum Gal-1 concentration (including malignancy [18], heart failure [19], and renal function [20]) were collected in detail, and further adjusted in the multivariate regression analysis.

**Fig 1:**
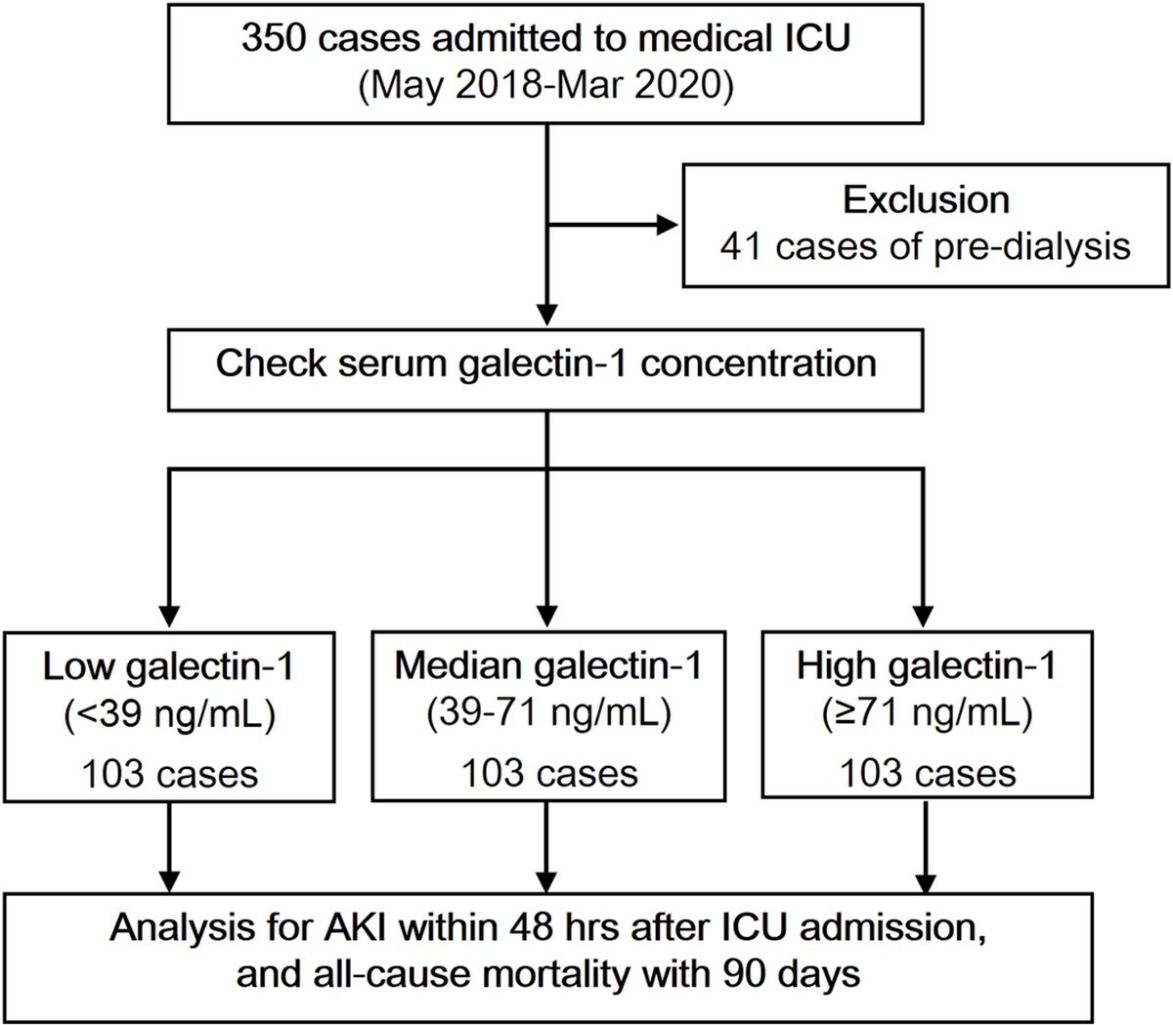
Flowchart of patient enrollment and classification. ICU, intensive care unit; AKI, acute kidney injury.

Trained personnel with data collection experience collected demographic and clinical data, including age, sex, body mass index (BMI), co-morbidities, drug exposure, etiologies of ICU admission, disease severity, and laboratory values at the time of ICU admission. Information on patients’ comorbidities, medication prescriptions, and etiologies of ICU admission was collected by detailed chart review. The Acute Physiology and Chronic Health Evaluation II (APACHE II) and Sequential Organ Failure Assessment (SOFA) scores were calculated to assess disease severity at the time of ICU admission [21, 22]. The lowest mean arterial pressure (MAP) and the use of inotropes/vasopressors within 24 h after ICU admission were recorded. Septic shock was defined as hypotension requiring vasopressors to maintain an MAP ≥ 65 mmHg and a serum lactate concentration > 18 mg/dL [14]. White blood cell counts and blood chemistry studies (determination of hemoglobin, glucose, lactate, and creatinine levels) were performed on ICU admission using routine laboratory methods. The estimated glomerular filtration rate (eGFR) was calculated using the Chronic Kidney Disease Epidemiology Collaboration equation [23]. A flowchart of patient enrollment is provided as **Fig 1**. This study was approved by the Research Ethics Committee of Taipei Veterans General Hospital and conducted according to the principles of the Declaration of Helsinki. All participants provided written informed consents.

### Serum Gal-1 measurement

Trained registered nurses obtained blood samples from participants within 24 h after ICU admission. Serum Gal-1 concentrations were determined using the commercially available Human Galectin-1 Quantikine ELISA Kit DGAL10 (R&D Systems, Inc., Minneapolis, MN, USA). The enrolled patients were grouped into tertiles according to their serum Gal-1 concentrations.

### Study outcomes and patient follow up

We retrieved data on primary and secondary outcomes from the hospital’s electronic medical records system. Primary outcomes were all-cause mortality in the ICU and at 90 days. Secondary outcomes were the durations of ICU admission and hospitalization, occurrence of AKI within 48 h after ICU admission, and dialysis dependency at the time of discharge. AKI was defined as the acute deterioration of renal function according to the Kidney Disease Improving Global Outcomes criteria [24, 25]. Patients with either stage 1, stage 2, or stage 3 AKI were all employed in the outcomes of this study. Dialysis dependency was defined as the requirement for renal replacement therapy (hemodialysis or peritoneal dialysis) after discharge due to irreversible kidney dysfunction [26].

### Statistical analysis

Categorical variables are presented as numbers and percentages and were assessed using the Chi-Squared test. Continuous variables are expressed as medians and interquartile ranges and were analyzed using the Kruskal–Wallis test. Kaplan–Meier survival curves and the log-rank test were used to estimate 90-day mortality. Area under receiver operating characteristic (ROC) curve was used to evaluate the accuracy of Gal-1 in predicting the incidence of 90-days mortality, and the criterion value was determined by the Youden index. Cox proportional-hazard regression analyses were used to calculate hazard ratios (HRs) and 95% confidence intervals (CIs) for factors associated with 90-day mortality. Variables with *p* values < 0.1 in the univariate regression analysis were entered into a final forward stepwise multivariate regression model. To confirm the independent association between Gal-1 concentration and 90-days mortality, we conducted another multivariate model adjusting for age, gender, heart failure, malignancy, etiologies of ICU admission, initial eGFR, and variables with *p* values < 0.05 in the univariate regression. Sensitivity analyses using different definitions of study exposure were also performed. Gal-1 concentrations was either presented as continuous variable; or divided by the criterion value of ROC curve, or by the Gal-1 value of reported viral [27] or bacterial [28] infection in the sensitivity analyses. To investigate the effect of Gal-1 modified by different conditions, we performed subgroup analyses with the cohort stratified by the presence of diabetes, proteinuria, initial eGFR, pneumonia, and septic shock. *P* values < 0.05 was considered to be statistically significant. All analyses were performed using SPSS software (version 19.0; IBM Corporation, Armonk, NY, USA).

## Results

### Baseline characteristics of study participants

In total, 309 patients admitted to the ICU with critical conditions were included in this study. The median age of the study population was 67 (range 57–78) years, and 68% of participants were male. Sepsis was the leading reason for ICU admission (87.4% prevalence) and pneumonia was the most common source of infection. The baseline characteristics of the study population are summarized in **Table 1**.

**Table 1:** Baseline characteristics of critically ill patients grouped by serum galectin-1 concentrations.

	Low galectin-1 (<39 ng/mL) n = 103	Median galectin-1 (39–71 ng/mL) n = 103	High galectin-1 (≥71 ng/mL) n = 103	*P*
Age (years)	64.0 (57.0–75.0)	68.0 (60.0–80.0)	68.0 (55.0–80.0)	0.185
Male gender	73 (70.9)	69 (67.0)	69 (67.0)	0.787
Body mass index	22.3 (19.7–24.9)	23.6 (20.8–26.6)	23.1 (20.1–27.0)	0.050
Hypertension	38 (36.9)	60 (58.3)	41 (39.8)	0.004
Diabetic mellitus	22 (21.4)	31 (30.1)	37 (35.9)	0.069
Heart failure	8 (7.8)	13 (12.6)	16 (15.5)	0.222
Cirrhosis	7 (6.8)	6 (5.8)	7 (6.8)	0.948
Malignancy (solid tumor)	42 (40.8)	35 (34.0)	38 (36.9)	0.599
ACEi / ARB exposure	17 (16.5)	25 (24.3)	23 (22.3)	0.363
Diuretics exposure	9 (8.7)	22 (21.4)	19 (18.4)	0.036
Nephrotoxic agents exposure	5 (4.9)	11 (10.7)	7 (6.8)	0.268
Etiologies of ICU admission				
Sepsis	83 (80.6)	91 (88.3)	96 (93.2)	0.023
Pneumonia	77 (74.8)	77 (74.8)	73 (70.9)	0.767
Acute heart failure	1 (1.0)	3 (2.9)	6 (5.8)	0.140
Massive bleeding	8 (7.8)	5 (4.9)	8 (7.8)	0.631
Disease severity				
APACHE II scores	22.0 (18.0–29.0)	26.0 (20.0–32.0)	29.0 (24.0–35.0)	<0.001
SOFA scores	8.0 (6.0–10.3)	10.0 (8.0–12.0)	11.0 (9.0–13.3)	<0.001
Ventilator usage	94 (91.3)	86 (83.5)	99 (96.1)	0.009
Inotrope/ vasopressor usage	55 (53.4)	46 (44.7)	66 (64.1)	0.020
Mean arterial pressure (mmHg)	55.3 (49.0–64.7)	58.5 (51.6–67.3)	52.3 (43.7–62.7)	0.004
Septic shock	17 (16.5)	19 (18.4)	37 (35.9)	0.001
White blood cells (K)	8.4 (4.6–12.2)	9.4 (5.1–15.5)	8.7 (3.7–14.1)	0.154
Hemoglobin (mg/dL)	9.5 (8.2–11.2)	9.0 (7.9–10.1)	8.2 (6.8–9.3)	<0.001
Initial eGFR (mL/min /1.73m^2^)	72.4 (38.3–93.7)	36.1 (21.3–65.4)	21.3 (13.8–37.2)	<0.001
Proteinuria	61 (59.2)	83 (80.6)	84 (81.6)	<0.001
Glucose (mg/dL)	142.0 (102.0–219.0)	130.5 (111.8–205.3)	129.5 (90.0–190.0)	0.234
Lactate, 0h (mg/dL)	10.8 (7.2–19.4)	9.9 (7.2–19.8)	18.9 (9.9–36.9)	0.001
Galectin-1 (ng/mL)	29.4 (24.3–34.8)	52.8 (47.5–60.6)	99.7 (82.7–148.2)	<0.001

ACEi, angiotensin-converting enzyme inhibitor; ARB, angiotensin receptor blocker; APACHE, Acute Physiology and Chronic Health Evaluation; SOFA, Sequential Organ Failure Assessment; eGFR, estimated glomerular filtration rate.

Patients with serum Gal-1 concentrations < 39 ng/ml were allocated to the low Gal-1 tertile, those with serum Gal-1 concentrations of 39–71 ng/ml were allocated to the median Gal-1 tertile, and those with serum Gal-1 concentrations > 71 ng/ml were allocated to the high Gal-1 tertile. The prevalence of sepsis, septic shock, and proteinuria and the percentage of patients requiring ventilators and inotropes/vasopressors were greater among patients in the high Gal-1 tertile than among those in the low and median Gal-1 tertiles. Patients in the high Gal-1 tertile also had higher APACHE II and SOFA scores, higher lactate concentrations, and lower hemoglobin levels, MAP, and eGFRs at ICU admission.

### Associations of the serum Gal-1 level with primary outcomes

**Fig 2:**
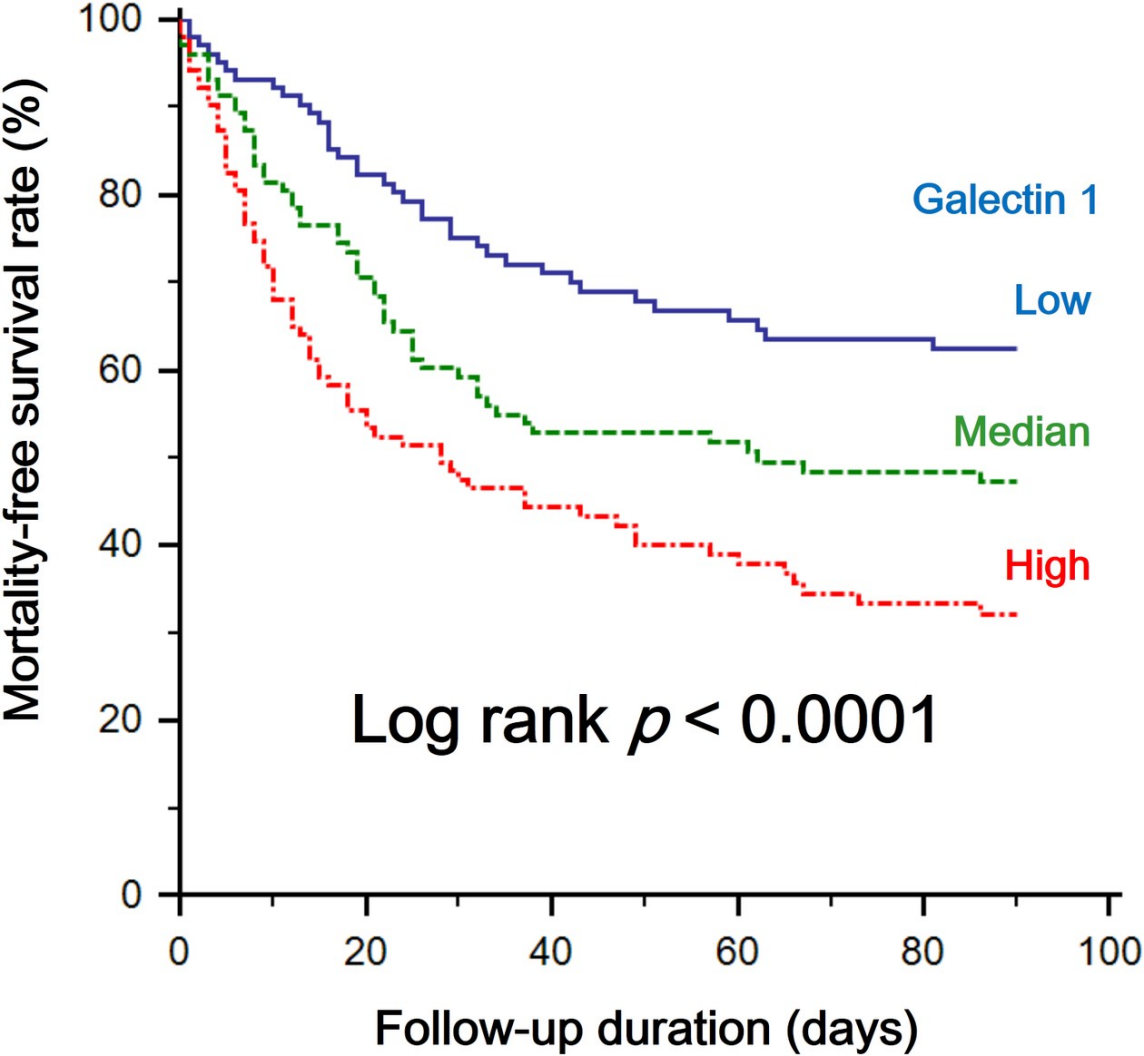
Kaplan–Meier curves of freedom from 90-day mortality in patients grouped by serum galectin-1 concentrations.

Participants’ clinical outcomes are summarized in **Table 2**. The ICU (42.7% vs. 30.1% and 17.5%; *p* < 0.001) and 90-day all-cause mortality rates (66% vs. 50.4% and 35.9%; *p* < 0.001) were greater in the high Gal-1 tertile than in the median and low Gal-1 tertiles. Accordingly, the 90-day survival rate was significantly lower in patients in the high Gal-1 tertile than in the other two groups (*p* < 0.0001; **Fig 2**). ROC curve characterizing the ability of the serum galectin-1 level to predict the incidence of 90-day all-cause mortality is presented in **S1 Fig**. The area under receiver ROC curve is 0.650, and the criterion value of Gal-1 concentration is 50.4 ng/mL.

**Table 2:** Clinical outcomes of critically ill patients grouped by serum galectin-1 concentrations.

	Low galectin-1 (<39 ng/mL) n = 103	Median galectin-1 (39–71 ng/mL) n = 103	High galectin-1 (≥71 ng/mL) n = 103	*P*
**Mortality during follow-up period**				
Length of ICU stay (days)	8.0 (5.0–14.0)	9.0 (6.0–18.0)	9.0 (5.0–14.0)	0.265
Length of hospitalization (days)	25.0 (14.0–38.0)	25.0 (12.0–38.0)	20.0 (9.0–44.0)	0.659
Mortality, in ICU	18 (17.5)	31 (30.1)	44 (42.7)	<0.001
Mortality, 90-days	37 (35.9)	52 (50.4)	68 (66.0)	<0.001
**AKI within 48 hours after ICU admission**				
AKI (total cases)	18 (17.5)	39 (37.9)	52 (50.5)	<0.001
Stage 1	10 (9.7)	20 (19.4)	15 (14.6)	<0.001
Stage 2–3	8 (7.8)	19 (18.4)	37 (35.9)	
Dialysis-dependence after discharge	0 (0)	5 (4.9)	7 (6.8)	0.034

AKI, acute kidney injury; ICU, intensive care unit.

Compared with patients in the low Gal-1 tertile, both patients in the median (HR 1.64, 95% CI 1.08–2.51, *p* = 0.021; **Table 3**) and in the high Gal-1 tertiles (HR 2.50, 95% CI 1.67–3.74, *p* < 0.001) were with greater HR of 90-days all-cause mortality. In the forward stepwise multivariate model, patients in the median (adjusted HR 2.11, 95% CI 1.24–3.60, *p* = 0.006) and in the high Gal-1 tertiles (adjusted HR 3.21, 95% CI 1.90–5.42, *p* < 0.001) were still associated with greater 90-day mortality after adjusting for the BMI, malignancy, sepsis, SOFA score, and lactate concentration. Even after adjusting for age, gender, BMI, hypertension, heart failure, malignancy, etiologies of ICU admission (including sepsis, pneumonia, acute heart failure, massive bleeding), APACHE II scores, SOFA scores, ventilator usage, inotrope/ vasopressor usage, MAP, septic shock, hemoglobin, initial eGFR, and lactate concentration, patients in the median (adjusted HR 2.56, 95% CI 1.44–4.55, *p* = 0.001; **S1 Table**) and in the high Gal-1 tertiles (adjusted HR 3.23, 95% CI 1.82–5.73, *p* < 0.001) remained independently associated with greater 90-day mortality. Sensitivity analyses using different definitions of study exposure also showed similar findings. Even if we presented Gal-1 value as a continuous variable (**S2 Table**), grouped patients according to the criterion value of ROC curve (**S3 Table**), or grouped by the serum Gal-1 values of reported viral (**S4 Table**) or bacterial infection (**S5 Table**), critically ill patients with higher Gal-1 levels were still significantly associated with greater 90-days mortality. Subgroup comparisons according to the serum Gal-1 level are shown in **Table 4**. High serum Gal-1 levels were associated with greater 90-day mortality than were low serum Gal-1 levels across different subgroups.

**Table 3:** Multivariate associations of the galectin-1 tertiles and factors with all-cause mortality within 90 days among critically ill patients (forward stepwise multivariate regression model).

	Univariate		Multivariate*	
	Crude HR (95% CI)	*P*	Adjusted HR (95% CI)	*P*
Galectin-1 concentration				
Low (<39 ng/mL)	Reference		Reference	
Median (39–71 ng/mL)	1.64 (1.08–2.51)	0.021	2.11 (1.24–3.60)	0.006
High (≥71 ng/mL)	2.50 (1.67–3.74)	<0.001	3.21 (1.90–5.42)	<0.001
Age	1.00 (0.99–1.01)	0.488		
Male gender	1.29 (0.92–1.83)	0.145		
Body mass index	0.96 (0.93–1.00)	0.041	0.94 (0.90–0.98)	0.006
Hypertension	0.63 (0.46–0.87)	0.005		
Diabetic mellitus	0.74 (0.52–1.06)	0.105		
Heart failure	0.82 (0.50–1.36)	0.436		
Cirrhosis	1.16 (0.61–2.21)	0.647		
Malignancy (solid tumor)	1.48 (1.08–2.03)	0.015	1.87 (1.27–2.75)	0.001
ACEi / ARB exposure	0.89 (0.60–1.32)	0.575		
Diuretics exposure	1.15 (0.76–1.74)	0.509		
Nephrotoxic agents exposure	0.89 (0.50–1.61)	0.706		
Etiologies of ICU admission				
Sepsis	1.99 (1.10–3.58)	0.023	2.95 (1.07–8.11)	0.036
Pneumonia	0.91 (0.64–1.30)	0.609		
Acute heart failure	0.52 (0.17–1.63)	0.264		
Massive bleeding	0.63 (0.31–1.27)	0.195		
Disease severity				
APACHE II scores	1.06 (1.04–1.09)	<0.001		
SOFA scores	1.16 (1.11–1.22)	<0.001	1.11 (1.05–1.18)	<0.001
Ventilator usage	2.23 (1.09–4.53)	0.028		
Inotrope/ vasopressor usage	1.69 (1.23–2.33)	0.001		
Mean arterial pressure (mmHg)	0.98 (0.97–0.99)	0.002		
Septic shock	1.95 (1.39–2.75)	<0.001		
White blood cells (K)	0.99 (0.97–1.01)	0.183		
Hemoglobin (mg/dL)	0.86 (0.79–0.93)	<0.001		
Initial eGFR (mL/min /1.73m2)	1.00 (0.99–1.00)	0.121		
Proteinuria	1.15 (0.80–1.65)	0.466		
Glucose (mg/dL)	1.00 (1.00–1.00)	0.270		
Lactate, 0h (mg/dL)	1.01 (1.01–1.02)	<0.001	1.01 (1.00–1.01)	0.048

* Adjusted for variables with *p* < 0.1 in the univariate analysis.

HR, hazard ratio; CI, confidence interval; ACEi, angiotensin-converting enzyme inhibitor; ARB, angiotensin receptor blocker; ICU, intensive care unit; APACHE, Acute Physiology and Chronic Health Evaluation; SOFA, Sequential Organ Failure Assessment; eGFR, estimated glomerular filtration rate.

**Table 4:** Stratified risk of 90-day mortality among patients grouped by the presence of diabetes, proteinuria, renal insufficiency, pneumonia, and septic shock.

Subgroup	Median galectin-1 group*		High galectin-1 group*		*P* for interaction
(events/ subjects)	Adjusted HR (95% CI)^✝^	*P*	Adjusted HR (95% CI)^✝^	*P*	
Diabetes mellitus					
Yes (40 / 90)	3.83 (0.92–15.90)	0.065	2.96 (0.78–11.23)	0.112	0.898
No (117 / 219)	1.83 (1.03–3.28)	0.041	3.41 (1.90–6.11)	<0.001	
Proteinuria					
Yes (119 / 228)	1.88 (1.00–3.54)	0.052	2.81 (1.51–5.23)	0.001	0.726
No (38 / 81)	3.69 (1.31–10.39)	0.014	3.40 (1.11–10.44)	0.032	
Initial eGFR <45					
Yes (88 / 172)	4.25 (1.58–11.48)	0.004	4.75 (1.80–12.48)	0.002	0.120
No (69 / 137)	1.59 (0.77–3.27)	0.210	2.83 (1.31–6.16)	0.008	
Pneumonia					
Yes (116 / 227)	2.07 (1.14–3.75)	0.017	3.33 (1.81–6.11)	<0.001	0.794
No (41 / 82)	3.44 (0.99–12.04)	0.053	3.74 (1.06–13.24)	0.041	
Septic shock					
Yes (48 / 73)	1.99 (0.64–6.24)	0.237	3.99 (1.48–10.74)	0.006	0.287
No (109 / 236)	2.17 (1.18–3.97)	0.013	2.56 (1.35–4.86)	0.004	

*Compared with the low galectin-1 group.

^✝^Adjusted for the body mass index, malignancy, sepsis, Sequential Organ Failure Assessment score, and lactate concentration.

HR, hazard ratio; CI, confidence interval; eGFR, estimated glomerular filtration rate.

### Associations of the serum Gal-1 level with secondary outcomes

The durations of ICU admission and hospitalization did not differ according to the serum Gal-1 concentration (**Table 2**). The incidences of AKI within 48 h after ICU admission (50.5% vs. 37.9% and 17.5%; *p* < 0.001) and dialysis dependency (6.8% vs. 4.9% and 0%; *p* = 0.034) were greater among patients in the high Gal-1 tertile than among those in the median and low tertiles. In multivariate analysis adjusted for the SOFA score and initial eGFR, the risk of AKI increased with the serum Gal-1 level (median vs. low: aOR 2.77, 95% CI 1.21–6.34, *p* = 0.016; high vs. low: aOR 2.88, 95% CI 1.20–6.88, *p* = 0.017; **Table 5**). Subgroup analysis revealed no significant difference in AKI according to the Gal-1 concentration (**Table 6**).

**Table 5:** Multivariate associations of the galectin-1 tertiles and factors with acute kidney injury within 48 h after intensive care unit admission (forward stepwise multivariate regression model).

	Univariate		Multivariate*	
	Crude OR (95% CI)	*P*	Adjusted OR (95% CI)	*P*
Galectin-1 concentration				
Low (<39 ng/mL)	Reference		Reference	
Median (39–71 ng/mL)	2.88 (1.51–5.49)	0.001	2.77 (1.21–6.34)	0.016
High (≥71 ng/mL)	4.82 (2.54–9.12)	<0.001	2.88 (1.20–6.88)	0.017
Age	1.00 (0.99–1.02)	0.558		
Male gender	1.04 (0.63–1.72)	0.884		
Body mass index	1.02 (0.97–1.08)	0.363		
Hypertension	1.67 (1.04–2.67)	0.032		
Diabetic mellitus	1.52 (0.92–2.52)	0.102		
Heart failure	1.66 (0.83–3.33)	0.151		
Cirrhosis	1.24 (0.49–3.14)	0.648		
Malignancy (solid tumor)	0.80 (0.49–1.31)	0.380		
ACEi / ARB exposure	1.40 (0.80–2.46)	0.236		
Diuretics exposure	1.27 (0.68–2.37)	0.446		
Nephrotoxic agents exposure	1.76 (0.75–4.13)	0.195		
Etiologies of ICU admission				
Sepsis	1.68 (0.79–3.59)	0.182		
Pneumonia	1.15 (0.68–1.97)	0.604		
Acute heart failure	0.78 (0.20–3.08)	0.723		
Massive bleeding	0.91 (0.36–2.33)	0.847		
Disease severity				
APACHE II scores	1.06 (1.03–1.09)	<0.001		
SOFA scores	1.23 (1.14–1.34)	<0.001	1.17 (1.06–1.29)	0.002
Ventilator usage	0.80 (0.37–1.73)	0.569		
Inotrope/ vasopressor usage	1.26 (0.79–2.02)	0.329		
Mean arterial pressure (mmHg)	0.99 (0.97–1.00)	0.098		
Septic shock	1.74 (1.02–2.97)	0.043		
White blood cells (K)	1.02 (1.00–1.04)	0.122		
Hemoglobin (mg/dL)	0.96 (0.86–1.07)	0.483		
Initial eGFR (mL/min /1.73m2)	0.97 (0.96–0.98)	<0.001	0.99 (0.98–1.00)	0.020
Proteinuria	2.13 (1.19–3.80)	0.011		
Glucose (mg/dL)	1.00 (0.99–1.00)	0.077		
Lactate, 0h (mg/dL)	1.01 (1.00–1.02)	0.015		

* Adjusted for variables with *p* < 0.1 in the univariate analysis.

OR, odds ratio; CI, confidence interval; ACEi, angiotensin-converting enzyme inhibitor; ARB, angiotensin receptor blocker; ICU, intensive care unit; APACHE, Acute Physiology and Chronic Health Evaluation; SOFA, Sequential Organ Failure Assessment; eGFR, estimated glomerular filtration rate.

**Table 6:** Stratified risk of acute kidney injury in patients grouped by the presence of diabetes, proteinuria, renal insufficiency, pneumonia, and septic shock.

Subgroup	Median galectin-1 group*		High galectin-1 group*		*P* for interaction
(events / subjects)	Adjusted OR (95% CI)^✝^	*P*	Adjusted OR (95% CI)^✝^	*P*	
Diabetes mellitus					
Yes (38 / 90)	2.72 (0.70–10.61)	0.149	2.80 (0.70–11.22)	0.147	0.948
No (71 / 219)	1.87 (0.77–4.56)	0.166	1.81 (0.72–4.58)	0.210	
Proteinuria					
Yes (90 / 228)	1.83 (0.78–4.26)	0.164	1.92 (0.81–4.58)	0.140	0.871
No (19 / 81)	2.46 (0.53–11.55)	0.253	2.42 (0.43–13.54)	0.313	
Initial eGFR <45					
Yes (87 / 172)	1.16 (0.45–3.02)	0.763	1.13 (0.43–2.95)	0.806	0.281
No (22 / 137)	4.69 (1.28–17.14)	0.020	3.68 (0.80–16.84)	0.093	
Pneumonia					
Yes (82 / 227)	1.88 (0.83–4.28)	0.133	2.42 (1.02–5.75)	0.045	0.821
No (27 / 82)	2.35 (0.44–12.60)	0.320	1.54 (0.28–8.36)	0.618	
Septic shock					
Yes (33 / 73)	3.50 (0.66–18.58)	0.142	1.73 (0.38–7.87)	0.480	0.773
No (76 / 236)	1.87 (0.82–4.30)	0.140	2.35 (0.96–5.73)	0.061	

*Compared with the low galectin-1 group.

^✝^Adjusted for Sequential Organ Failure Assessment score and initial eGFR.

OR, odds ratio; CI, confidence interval; eGFR, estimated glomerular filtration rate.

## Discussion

To our knowledge, this study is the first investigation of associations of the Gal-1 level with all-cause mortality and AKI in critically ill patients. It demonstrated that higher serum Gal-1 concentrations increase the risk of mortality in the ICU and at 90 days. This increase remained significant after controlling for the body mass index, malignancy, sepsis, SOFA score, and serum lactate level and was consistent across subgroups. The incidences of AKI and dialysis dependency after discharge were also greater in the high Gal-1 group than in the low Gal-1 group. In multivariate analysis, the serum Gal-1 concentration remained an independent risk factor for AKI after controlling for the SOFA score and initial renal function. These findings provide indirect evidence for Gal-1’s involvement in acute infection or inflammation.

Gal-1 is the first protein identified in the galectin family, abundant in muscles, neurons, thymus, kidney, and placenta [29, 30]. Gal-1 is mainly synthesized in the immune system, and secreted by the macrophages, activated T and B cells, and regulatory T cells [31]. Gal-1 has immunosuppressive and anti-inflammatory effects [32, 33]. It promotes the apoptosis of CD8, Th1, and Th17 lymphocytes, which enhances resolution of inflammation, compromises the anti-microbial immune response, and favors tumor-immune escape [31]. Elevated serum Gal-1 concentration is reported in various infectious diseases, including viral [7], bacterial [8], and parasite infection [9]. Elevated Gal-1 concentration is also observed in chronic inflammation and autoimmune disorders, such as rheumatoid arthritis [34] and inflammatory bowel disease [35]. Gal-1 also plays an essential role in cardiovascular disease, which prevents inflammation-induced neurodegeneration and improves neurogenesis after ischemic stroke [36]. Gal-1 is secreted early after myocardial infarction, thereby enhancing the resolution of cardiac inflammation. In an experimental study, mice lacking Gal-1 showed deteriorated ventricular remodeling after acute myocardial infarction [5]. Finally, Gal-1 is involved in the progression of cancer. Secreted Gal-1 mediates cell aggregation and adhesion to the extracellular matrix, which are crucial for tumor invasion of surrounding tissues [37]. It helps tumors to escape immunosurveillance [38]. High Gal-1 expression is reported to be associated with poor prognosis in several types of digestive cancers [39].

Numerous literatures support the diagnostic and prognostic significance of Gal-1 in infectious diseases [7, 8]. Recent reviews even suggested the immune-regulatory functions of galectins (mainly Gal-1, galectin-3, and galectin-9) as potential therapeutic strategies for viral infection, especially for influenza A virus [10]. Compared to other pro-inflammatory biomarkers, such as C-reactive protein or galectin-3, the anti-inflammatory nature of Gal-1 makes itself a promising therapeutic target for the overwhelming host response in acute infection. Intranasal treatment with recombinant Gal-1 reduced virus replication, inflammation, and apoptosis in lung; and further enhances the survival of mice with influenza A virus infection [40]. In another animal study, intravenous injection of Gal-1, but not galectin-3, inhibited leukocyte recruitment into the peritoneal cavity in rats with experimental peritonitis [41].

Despite the experimental studies investigating Gal-1’s role in infectious disease, evidence support the clinical relevance of Gal-1 in septic patients is very limited. Sepsis remains a significant challenge in medicine and is the most common cause of death in critically ill patients admitted to the ICU [42]. SIRS, another common reason for ICU admission, is a clinical syndrome that results from deregulation of the inflammatory response to infections or non-infectious insults, such as acute pancreatitis [43]. Biomarkers have been developed to diagnose, predict, or influence the disease severity of sepsis or SIRS. Our study revealed the risk of mortality increased with the serum Gal-1 concentration in critically ill patients. Nearly 90% of the patients in this study were with sepsis, and all the enrolled patients were with SIRS while admitted to ICU. Our study suggests Gal-1 may be a novel prognostic predictor for patients with sepsis or SIRS. To investigate the difference between patients with and without acute illness, we compared the Gal-1 concentrations of patients admitted to the ICU with those admitted for coronary angiography (CAG) and without obstructive coronary artery disease, which was measured in our previous study [20]. Gal-1 concentrations were significantly higher among patients admitted to ICU than among those admitted for CAG (52.8 *vs*. 17.4 ng/mL, *p* < 0.001; **S2 Fig**). Serum Gal-1 levels may reflect the severity of infection / inflammation and the host response to it. A complicated and overwhelming host response leads to multiorgan dysfunction [44], thereby increasing mortality [45].

We had adjusted following confounding factors that may affect serum Gal-1 concentrations in the presented study. First, serum Gal-1 level can be affected by renal function. AKI occurs in up to 36–67% of critically ill patients during their ICU stays [46, 47], and is associated with increased morbidity, mortality, and duration of hospitalization [48]. Its causes include sepsis, inflammation, hepatorenal and cardiorenal syndromes, and nephrotoxic agents. Gal-1 was reported to regulate podocin production and podocyte damage [49]. Gal-1 elevation has been associated with diabetic nephropathy [50] and renal function decline [20]. Hence it is reasonable that our study revealed significant associations between serum Gal-1 levels, initial eGFR, and incidence of AKI during ICU stay (**Table 5**). Our findings further strengthen the association of Gal-1 with AKI. Another confounding factor is the prevalence of cancer. Gal-1 is an essential mediator of tumor-immune escape [51]. The Gal-1 secreted from tumor cell help it to evade immune responses. Corapi *et al*. [38] found that the absence of Gal-1 in the T lymphocytes of patients with prostate cancer potentiated anti-tumor immune responses. High Gal-1 expression is a predictor of poor prognosis in cancer population [39]. Since patients with solid tumors comprised 37% of our study population, their influence on Gal-1 concentrations and outcomes should be evaluated carefully. Patients with cardiovascular diseases were also linked with elevated serum Gal-1 concentration [19]. Though our cohort did not included cases with acute myocardial infarction or acute stroke, about 12% of the enrolled patients were with heart failure. In the multivariate model intentionally adjusting for initial eGFR, malignancy, and heart failure, patients with high Gal-1 concentrations remained independently associated with greater 90-day mortality (**S1 Table**).

## Limitations

This study has several limitations. First, it was conducted at a single-center with relatively few patients. Second, given the lack of a reference Gal-1 range, we could only divide patients into tertiles according to serum Gal-1 concentrations. Nevertheless, sensitivity analyses using different definitions of study exposure (**S2–S5 Tables**) also showed significant association between Gal-1 value and 90-days mortality. Third, co-morbidities and disease severity differed significantly among groups, indicting the presence of selection bias. Although we performed adjusted analyses, some unmeasured confounding factors may not have been accounted for. Finally, the study was observational and the causality of relationships could not be established.

## Conclusions

Elevation of the serum Gal-1 concentration was associated with high ICU and 90-day mortality rates. The risks of AKI were also increased in patients with high Gal-1 levels. Serum Gal-1 may be a prognostic predictor for critically ill population. Further large-scale study is need to clarify the clinical significance of Gal-1 in patients with sepsis or acute inflammation.

## Supporting information

S1 Fig ROC curve of serum Gal-1 value in prediction the 90-days mortality. (DOCX)

S2 Fig Comparison of serum Gal-1 value between the ICU patients and control. (DOCX)

S1 Dataset All relevant data are within the paper and its supporting information files. (XLSX)

S1 Table Sensitivity analysis 1. Intentionally adjusted for age, gender, heart failure, malignancy, etiologies of ICU admission, initial eGFR, and variables with *p* < 0.05 in the univariate regression.
(DOCX)

S2 Table Sensitivity analysis 2. Ga-1 presented as continuous variable.
(DOCX)

S3 Table Sensitivity analysis 3. Allocated by the Gal-1 criterion value of ROC curve.
(DOCX)

S4 Table Sensitivity analysis 4. Allocated by the reported Gal-1 value of viral infection.
(DOCX)

S5 Table Sensitivity analysis 5. Allocated by the reported Gal-1 value of bacterial infection.
(DOCX)
