# Supplementary material for: Elevated serum galectin-1 concentrations are associated with increased risks of mortality and acute kidney injury in critically ill patients
Source: PLoS One. 2021 Sep 24;16(9):e0257558. doi: 10.1371/journal.pone.0257558 (PMC8462742; doi:10.1371/journal.pone.0257558)
Supplement: S1 Fig — (DOCX) [file pone.0257558.s001.docx]

**S1 Figure.** The c-statistics and ROC curve of circulating galectin-1 concentration in prediction the 90-days mortality of critically ill patients admitted to intensive care unit.


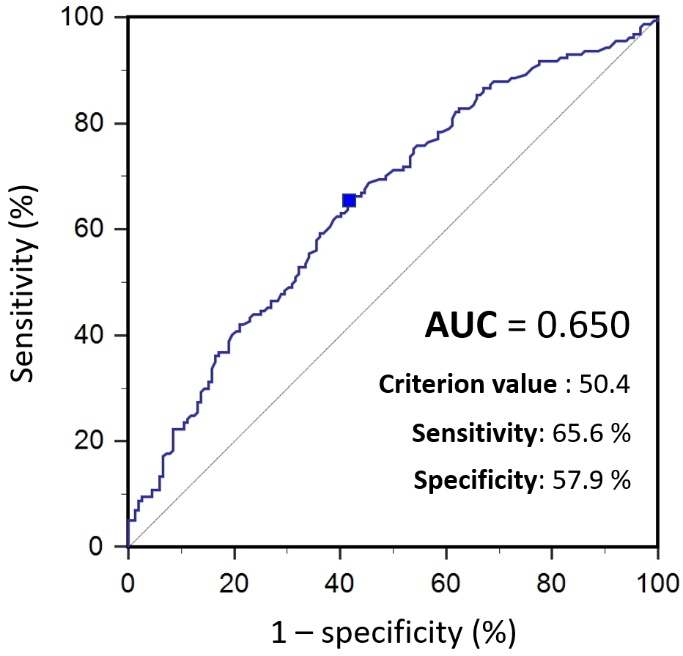


ROC curve, receiver operating characteristic curve; AUC, area under ROC curve
