# Supplementary material for: Elevated serum galectin-1 concentrations are associated with increased risks of mortality and acute kidney injury in critically ill patients
Source: PLoS One. 2021 Sep 24;16(9):e0257558. doi: 10.1371/journal.pone.0257558 (PMC8462742; doi:10.1371/journal.pone.0257558)
Supplement: S2 Fig — (DOCX) [file pone.0257558.s002.docx]

**S2 Figure.** Comparison of serum galectin-1 concentrations between critically ill patients admitted to ICU (ICU group) and patients without obstructive CAD (control group).


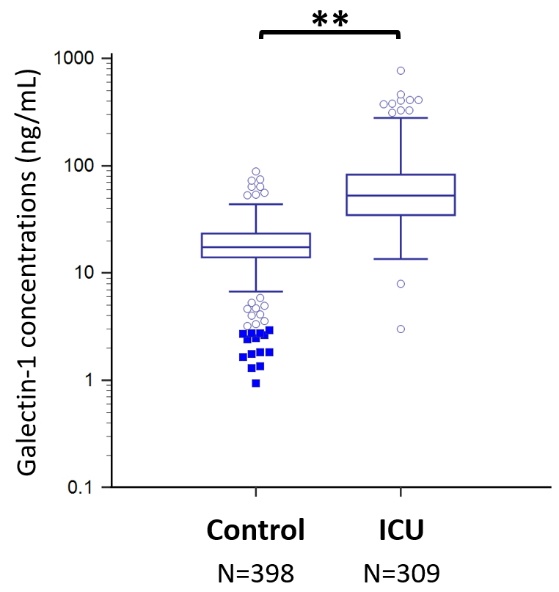


****p* < 0.001.

ICU, intensive care unit, CAD, coronary artery disease.
