# Supplementary material for: Elevated serum galectin-1 concentrations are associated with increased risks of mortality and acute kidney injury in critically ill patients
Source: PLoS One. 2021 Sep 24;16(9):e0257558. doi: 10.1371/journal.pone.0257558 (PMC8462742; doi:10.1371/journal.pone.0257558)
Supplement: S2 Table — Ga-1 presented as continuous variable. (DOCX) [file pone.0257558.s005.docx]

**S2 Table.** Multivariate associations of the galectin-1 concentration (presented as continuous variable) and factors with all-cause mortality within 90 days among critically ill patients.

|  | **Univariate Multivariate*** | | | | |
| --- | --- | --- | --- | --- | --- |
|  | **Crude HR (95% CI)** | ***P*** |  | **Adjusted HR (95% CI)** | ***P*** |
| Galectin-1 concentration (ng/mL) | 1.00 (1.00-1.01) | <0.001 |  | 1.00 (1.00-1.01) | 0.014 |
| Age | 1.00 (0.99-1.01) | 0.488 |  | 1.00 (0.99-1.02) | 0.786 |
| Male gender | 1.29 (0.92-1.83) | 0.145 |  | 1.31 (0.83-2.07) | 0.249 |
| Body mass index | 0.96 (0.93-1.00) | 0.041 |  | 0.96 (0.92-1.01) | 0.103 |
| Hypertension | 0.63 (0.46-0.87) | 0.005 |  | 0.75 (0.48-1.18) | 0.211 |
| Diabetic mellitus | 0.74 (0.52-1.06) | 0.105 |  |  |  |
| Heart failure | 0.82 (0.50-1.36) | 0.436 |  | 1.13 (0.57-2.56) | 0.727 |
| Cirrhosis | 1.16 (0.61-2.21) | 0.647 |  |  |  |
| Malignancy (solid tumor) | 1.48 (1.08-2.03) | 0.015 |  | 1.51 (0.98-2.32) | 0.060 |
| ACEi / ARB exposure | 0.89 (0.60-1.32) | 0.575 |  |  |  |
| Diuretics exposure | 1.15 (0.76-1.74) | 0.509 |  |  |  |
| Nephrotoxic agents exposure | 0.89 (0.50-1.61) | 0.706 |  |  |  |
| Etiologies of ICU admission |  |  |  |  |  |
| Sepsis | 1.99 (1.10-3.58) | 0.023 |  | 4.69 (1.57-14.02) | 0.006 |
| Pneumonia | 0.91 (0.64-1.30) | 0.609 |  | 0.49 (0.29-0.84) | 0.009 |
| Acute heart failure | 0.52 (0.17-1.63) | 0.264 |  | 1.38 (0.28-6.82) | 0.694 |
| Massive bleeding | 0.63 (0.31-1.27) | 0.195 |  | 0.40 (0.14-1.11) | 0.079 |
| Disease severity |  |  |  |  |  |
| APACHE II scores | 1.06 (1.04-1.09) | <0.001 |  | 1.03 (0.99-1.06) | 0.112 |
| SOFA scores | 1.16 (1.11-1.22) | <0.001 |  | 1.09 (1.01-1.18) | 0.036 |
| Ventilator usage | 2.23 (1.09-4.53) | 0.028 |  | 1.03 (0.40-2.70) | 0.950 |
| Inotrope/ vasopressor usage | 1.69 (1.23-2.33) | 0.001 |  | 1.12 (0.68-1.85) | 0.655 |
| Mean arterial pressure (mmHg) | 0.98 (0.97-0.99) | 0.002 |  | 1.00 (0.98-1.01) | 0.503 |
| Septic shock | 1.95 (1.39-2.75) | <0.001 |  | 0.64 (0.35-1.17) | 0.148 |
| White blood cells (K) | 0.99 (0.97-1.01) | 0.183 |  |  |  |
| Hemoglobin (mg/dL) | 0.86 (0.79-0.93) | <0.001 |  | 0.92 (0.83-1.03) | 0.134 |
| Initial eGFR (mL/min /1.73m2) | 1.00 (0.99-1.00) | 0.121 |  | 1.00 (1.00-1.01) | 0.464 |
| Proteinuria | 1.15 (0.80-1.65) | 0.466 |  |  |  |
| Glucose (mg/dL) | 1.00 (1.00-1.00) | 0.270 |  |  |  |
| Lactate, 0h (mg/dL) | 1.01 (1.01-1.02) | <0.001 |  | 1.01 (1.00-1.02) | 0.030 |

*Adjusted for age, gender, heart failure, malignancy, etiologies of ICU admission (including sepsis, pneumonia, acute heart failure, massive bleeding), initial eGFR, and variables with *p* < 0.05 in the univariate analysis.
