# Supplementary material for: Elevated serum galectin-1 concentrations are associated with increased risks of mortality and acute kidney injury in critically ill patients
Source: PLoS One. 2021 Sep 24;16(9):e0257558. doi: 10.1371/journal.pone.0257558 (PMC8462742; doi:10.1371/journal.pone.0257558)
Supplement: S5 Table — Allocated by the reported Gal-1 value of bacterial infection. (DOCX) [file pone.0257558.s008.docx]

**S5 Table.** Multivariate associations of high galectin-1 concentration [≥119.9 ng/ml, defined by the reported serum galectin-1 value of patients with bacterial (*Tropheryma whipplei*) infection] and factors with all-cause mortality within 90 days among critically ill patients.

|  | **Univariate Multivariate*** | | | | |
| --- | --- | --- | --- | --- | --- |
|  | **Crude HR (95% CI)** | ***P*** |  | **Adjusted HR (95% CI)** | ***P*** |
| Galectin-1 concentration |  |  |  |  |  |
| Low (<119.9 ng/mL) | Reference |  |  | Reference |  |
| High (≥119.9 ng/mL) | 2.50 (1.67-3.74) | <0.001 |  | 1.98 (1.08-3.63) | 0.027 |
| Age | 1.00 (0.99-1.01) | 0.488 |  | 1.00 (0.99-1.02) | 0.674 |
| Male gender | 1.29 (0.92-1.83) | 0.145 |  | 1.26 (0.80-1.99) | 0.323 |
| Body mass index | 0.96 (0.93-1.00) | 0.041 |  | 0.96 (0.92-1.01) | 0.100 |
| Hypertension | 0.63 (0.46-0.87) | 0.005 |  | 0.73 (0.47-1.13) | 0.159 |
| Diabetic mellitus | 0.74 (0.52-1.06) | 0.105 |  |  |  |
| Heart failure | 0.82 (0.50-1.36) | 0.436 |  | 1.22 (0.62-2.41) | 0.572 |
| Cirrhosis | 1.16 (0.61-2.21) | 0.647 |  |  |  |
| Malignancy (solid tumor) | 1.48 (1.08-2.03) | 0.015 |  | 1.49 (0.97-2.29) | 0.069 |
| ACEi / ARB exposure | 0.89 (0.60-1.32) | 0.575 |  |  |  |
| Diuretics exposure | 1.15 (0.76-1.74) | 0.509 |  |  |  |
| Nephrotoxic agents exposure | 0.89 (0.50-1.61) | 0.706 |  |  |  |
| Etiologies of ICU admission |  |  |  |  |  |
| Sepsis | 1.99 (1.10-3.58) | 0.023 |  | 5.00 (1.68-14.84) | 0.004 |
| Pneumonia | 0.91 (0.64-1.30) | 0.609 |  | 0.46 (0.27-0.77) | 0.003 |
| Acute heart failure | 0.52 (0.17-1.63) | 0.264 |  | 1.61 (0.33-1.88) | 0.557 |
| Massive bleeding | 0.63 (0.31-1.27) | 0.195 |  | 0.44 (0.16-1.19) | 0.105 |
| Disease severity |  |  |  |  |  |
| APACHE II scores | 1.06 (1.04-1.09) | <0.001 |  | 1.03 (1.00-1.07) | 0.080 |
| SOFA scores | 1.16 (1.11-1.22) | <0.001 |  | 1.08 (1.00-1.17) | 0.053 |
| Ventilator usage | 2.23 (1.09-4.53) | 0.028 |  | 1.02 (0.39-2.65) | 0.976 |
| Inotrope/ vasopressor usage | 1.69 (1.23-2.33) | 0.001 |  | 1.16 (0.70-1.92) | 0.564 |
| Mean arterial pressure (mmHg) | 0.98 (0.97-0.99) | 0.002 |  | 1.00 (0.98-1.01) | 0.518 |
| Septic shock | 1.95 (1.39-2.75) | <0.001 |  | 0.72 (0.40-1.29) | 0.267 |
| White blood cells (K) | 0.99 (0.97-1.01) | 0.183 |  |  |  |
| Hemoglobin (mg/dL) | 0.86 (0.79-0.93) | <0.001 |  | 0.91 (0.82-1.02) | 0.098 |
| Initial eGFR (mL/min /1.73m2) | 1.00 (0.99-1.00) | 0.121 |  | 1.00 (1.00-1.01) | 0.606 |
| Proteinuria | 1.15 (0.80-1.65) | 0.466 |  |  |  |
| Glucose (mg/dL) | 1.00 (1.00-1.00) | 0.270 |  |  |  |
| Lactate, 0h (mg/dL) | 1.01 (1.01-1.02) | <0.001 |  | 1.01 (1.00-1.02) | 0.072 |

*Adjusted for age, gender, heart failure, malignancy, etiologies of ICU admission (including sepsis, pneumonia, acute heart failure, massive bleeding), initial eGFR, and variables with *p* < 0.05 in the univariate analysis.
